# Gut microbiota in preterm infants with late-onset sepsis and pneumonia: a pilot case-control study

**DOI:** 10.1186/s12866-024-03419-w

**Published:** 2024-07-22

**Authors:** Ye Ma, Xiaoming Peng, Juan Zhang, Yulian Zhu, Ruiwen Huang, Guinan Li, Yunqin Wu, Changci Zhou, Jiajia You, Siwei Fang, Shiting Xiang, Jun Qiu

**Affiliations:** 1 Department of Neonatology, The Affiliated Children’s Hospital of Xiangya School of Medicine, Central South University (Hunan Children’s Hospital), 86 Ziyuan Road, Yuhua District, Changsha, China; 2 Department of Pediatrics, Zhuzhou Central Hospital, 116 Changnan Road, Tianyuan District, Zhuzhou, China; 3 Department of Obstetrics, Hunan Prevention and Treatment Institute for Occupational Diseases, 162 Xinjian West Road, Yuhua District, Changsha, China; 4 The School of Pediatrics, Hengyang Medical School, University of South China (Hunan Children’s Hospital), 28 West Changsheng Road, Zhengxiang District, Hengyang, China; 5 Pediatrics Research Institute of Hunan Province, The Affiliated Children’s Hospital of Xiangya School of Medicine, Central South University (Hunan Children’s Hospital), 86 Ziyuan Road, Yuhua District, Changsha, China

**Keywords:** Intestinal, Neonatal microbiome, Preterm infant, Late-onset sepsis, Diagnostic biomarkers

## Abstract

**Background:**

Late-onset sepsis (LOS) and pneumonia are common infectious diseases, with high morbidity and mortality in neonates. This study aimed to investigate the differences in the gut microbiota among preterm infants with LOS, or pneumonia, and full-term infants. Furthermore, this study aimed to determine whether there is a correlation between intestinal pathogenic colonization and LOS.

**Methods:**

In a single-center case‒control study, 16 S rRNA gene sequencing technology was used to compare gut microbiota characteristics and differences among the LOS group, pneumonia group, and control group.

**Results:**

Our study revealed that the gut microbiota in the control group was more diverse than that in the LOS group and pneumonia group (*P* < 0.05). No significant differences in diversity were detected between the LOS and pneumonia groups (*P >* 0.05). Compared with the control group, the abundances of *Akkermansia*, *Escherichia/Shigella*, and *Enterococcus* increased, while the abundances of *Bacteroides* and *Stenotrophomonas* decreased in the LOS and pneumonia groups. The pathogenic bacteria in infants with LOS were consistent with the distribution of the main bacteria in the intestinal microbiota. An increase in *Escherichia/Shigella* abundance may predict a high risk of LOS occurrence, with an area under the curve (AUC) of 0.773.

**Conclusion:**

Changes in the gut microbiota composition were associated with an increased risk of LOS and pneumonia. The dominant bacteria in the gut microbiota of the LOS group were found to be associated with the causative pathogen of LOS. Moreover, preterm infants exhibiting an elevated abundance of *Escherichia/Shigella* may be considered potential candidates for predicting the onset of LOS.

**Supplementary Information:**

The online version contains supplementary material available at 10.1186/s12866-024-03419-w.

## Introduction

Late-onset sepsis (LOS) is defined as sepsis occurring 72 h or more after birth [1]. LOS remains a challenge in neonatology, with an incidence of 1–4 cases of bacterial sepsis per 1000 live births and a mortality rate of 12% [2, 3]. Neonatal pneumonia is defined as a respiratory infection affecting the lung parenchyma that occurs within the first 28 days of life [4]. The morbidity rate of neonatal pneumonia ranges from 3.5 to 25%, and it contributes to 14% of all deaths among children under 5 years old [5, 6]. LOS and pneumonia are the most common causes of neonatal mortality due to infection [2, 3, 7]. Over the past few decades, the management of LOS and pneumonia, involving antibiotics and symptomatic supportive care, has undergone limited advancements [8]. When neonatal infection is suspected, it is difficult to make accurate and rapid microbial diagnoses by using clinical manifestations and laboratory results [9].Therefore, early and accurate diagnostic methods for LOS and pneumonia are critical for decreasing neonatal mortality.

Despite extensive research, the precise biological mechanisms underlying LOS remain unclear. There are two main hypotheses for the development of LOS. The first suggests that bacteria invade the bloodstream and cause LOS when mucosal integrity is compromised and immune defenses are impaired. The second hypothesis suggests that certain highly invasive bacterial strains can overcome host defenses. This is particularly true in preterm infants, where compromised tight junctions in the intestinal epithelium may allow bacterial ‘leakage’ into the bloodstream, potentially leading to gut-derived sepsis [10–13]. Several studies have shown that the intestinal leakage may be the cause of LOS [14, 15], but further studies are required to investigate the changes in the intestinal microbiome following the onset of sepsis. Disturbance of the gut microbiota not only affects the intestinal tract and contributes to LOS but also impacts distant organs such as the brain, liver, and lungs [16–18]. Inflammation and the gut microbiota play crucial roles in the transition between host health and disease [19]. The crosstalk between the gut microbiota and lungs, termed the “gut–lung axis,” is vital for the immune response and airway homeostasis [17, 20, 21]. Research has shown that the gut microbiota plays a protective role in the defense of the host against pneumonia [17]. During the neonatal period, exposure to intestinal symbiotic bacteria induces immunity against pulmonary infections in host lungs [17, 20], but the composition of the pneumonia microbial community has been poorly explored.

Currently, blood culture is used as the accepted standard for sepsis detection. However, this approach is time-consuming and has a low positive rate [3]. The classification of fecal and blood bacteria suggested that neonatal ecological dysbiosis may lay the foundation for LOS, which offers the possibility of early pathogen surveillance. However, previous studies have focused primarily on the intestinal microbiota before the onset of LOS and the examination related to the subsequent blood infection by these strains, which is a resource-intensive and impractical approach. From a clinical perspective, infants are typically tested for infection only when infection is suspected, highlighting the clinical significance of evaluating changes in the gut microbiota post infection. Therefore, this study aimed to screen for the presence of pathogenic bacterial colonization in the intestinal tract of premature infants with LOS, premature infants with pneumonia, and a control group using 16 S rRNA gene sequencing technology. The resulting data will provide initial evidence for potential early biomarkers of LOS. The findings of this study will establish a scientific basis for the early identification of pathogens in clinical practice.

## Methods

### Participants

We included 8 neonates with laboratory-confirmed LOS and 8 neonates with pneumonia who were admitted to the Neonatology Department of Human Children’s Hospital between August 2018 and October 2019 as participants in our study. Infants with pneumonia were matched with infants with LOS at a ratio of 1:1 according to gestational age (± one week) and birth weight. We selected 8 healthy full-term infants born at the Department of Obstetrics at the Hunan Prevention and Treatment Institute for Occupational Diseases from July 2022 to August 2022 with meconium samples as the control group. Ultimately, 8 premature infants experienced 8 invasive bacterial infections, and these patients were categorized into the LOS group (L). Eight preterm infants developed pneumonia, and these patients were categorized into the neonatal pneumonia group (P). Healthy full-term newborns were categorized into the control group (C). None of the patients received probiotics, and none of them had central venous catheters. Early-onset sepsis (EOS) (positive blood cultures at 72 h after birth), Bell’s stage 2 A necrotizing enterocolitis (NEC) or above, congenital gastrointestinal malformations, spontaneous intestinal perforations, fewer than two fecal samples available with a minimum weight of 100 mg, and missing or incomplete medical records were exclusion criteria for the LOS and pneumonia groups.

An infant with one or more symptoms was deemed to have LOS if a positive blood culture was obtained after three days of age, accompanied by positive results for the following indicators: temperature instability, leukocytosis or neutropenia, an elevated immature to total (I/T) neutrophil ratio, or an elevated C-reactive protein [1].

Pneumonia is an infection of the lower respiratory tract, involving the lung parenchyma. Late-onset pneumonia is often caused by pathogens encountered in the postnatal environment, either in the community (community-associated pneumonia), or in the hospital (hospital-associated pneumonia) [4].

Neonates included in the control group met the following criteria: no prenatal use of antibiotics, the absence of symptoms such as dyspnea, cyanosis, and poor postnatal responsiveness, and the ability to stay in the mother’s room.

### Ethics

Before initiating the study, ethical approval was obtained from the Hunan Children’s Hospital Ethics Committee (HCHLL- 2023-87, HCHLL − 2023-88, No. HCHLL-2020-53). In addition, written informed consent was obtained from the parents, legal guardians, or both for all the enrolled children, thus ensuring compliance with ethical standards.

#### Patient data

Information related to newborn hospitalizations was extracted from electronic medical records. Data related to birth weight, sex, age, gestational age, mode of delivery, use of antibiotics, feeding status, maternal age at pregnancy, clinical diagnosis, and laboratory parameters such as the white blood cell (WBC) count, neutrophil ratio (NR), blood platelet count (Plc), C-reactive protein (CRP) level, and procalcitonin (PCT) level prior to diagnosis were collected for each newborn.

#### Sample collection

Fecal samples were collected from diapers using a stool collection kit provided by Genesky Biotechnologies Inc., Shanghai, 201,315 (China). For a more detailed description, please refer to the Supplemental Material. Fecal samples were collected fresh and immediately frozen in an ice box. Then the samples were transported to the laboratory within 30 min and stored at − 80 °C.

### High-throughput 16 S rRNA gene sequencing and DNA extraction

16 S rRNA amplicon sequencing was performed by Genesky Biotechnologies Inc., Shanghai, 201,315 (China). A QIAamp Fast DNA Stool Mini Kit (QIAGEN ART.NO.56,104) was used to extract total genomic DNA. The integrity of the genomic DNA was evaluated through agarose gel electrophoresis, and the concentration and purity of the genomic DNA were determined through a Nanodrop 2000 spectrophotometer and a Qubit 3.0 spectrophotometer. The V4–V5 hypervariable regions of the 16 S rDNA gene were amplified with the primers 515 F (5′-GTGCCAGCMGCCGCGG-3′) and 907R (5′-CCGTCAATTCMTTTR AGTTT-3′) was amplified with [22] and then sequenced using the Illumina NovaSeq 6000 platform [23]. The sequencing data were stored in the NCBI Sequencing Read Archive under accession ID PRJNA926124.

### Gut microbiota analysis

The raw read sequences were further filtered to remove adapter sequences, primers, and low-quality reads by QIIME2 and the cutadapt plugin, thus improving the accuracy of later analysis [24]. The filtered sequences were clustered into operational taxonomic units (OTUs) with a similarity ≥ 97%, and the sequence with the highest abundance within each cluster was considered to be representative [25]. The sample species accumulation curve was analyzed using QIIME2 to comprehensively assess the sample content. Alpha diversity was evaluated through abundance and diversity index. Abundance was represented by the Chao 1 index, while diversity was indicated by Shannon indices. To assess the community composition and structure of the gut microbiota, principal coordinates analysis (PCoA) was employed for β diversity, utilizing the Bray Curtis distance calculated with QIIME2. Through the linear discriminant analysis (LDA) histogram and the cladogram, LDA effect size (LEfSe) analysis was used to identify species with significant differences in abundance among the LOS group, pneumonia group, and control group [26]. The differences in the relative abundance of the gut microbiota at the phylum and genus levels were used to evaluate the differences in the gut microbiota composition among LOS, pneumonia, and control groups.

### Statistical analysis

Statistical analysis was conducted using SPSS version 26 and R software (version 4.3.1). Normally distributed continuous variables are presented as the mean ± standard deviation (X̅ ± SD). Differences were compared using an independent t test, or one-way analysis of variance (ANOVA) when appropriate. Nonnormally distributed variables are expressed as medians and interquartile ranges [M (P25, P75)]. Differences were evaluated using the Wilcoxon rank-sum test or the Kruskal‒Wallis rank-sum test. Categorical data are presented as percentages and were evaluated using the chi-square test. A p value less than 0.05 was considered to indicate statistically significant. The ROC curve calculated and displayed with R software (version 4.3.1) was used to assess effective biomarkers for LOS.

## Results

### Clinical characteristics

A comprehensive summary of the clinical data is shown in Table 1. The gestational age and birth weight of the LOS group and pneumonia group were significantly lower than those of the control group. However, there were no significant differences among the three groups in terms of sex, mode of delivery, or the proportion of breastfed neonates. The premature infants included in the study all received antibiotics (Supplementary Table 1). None of the included preterm infants or full terms received probiotics.

**Table 1:** Clinical information comparison among preterm infant with LOS, preterm infant with pneumonia, and healthy full‒term infants

Variables	Preterm infants with LOS, *N* = 8.	Preterm infants with pneumonia, *N* = 8.	Control group, *N* = 8.	t/χ^2^ (#)	*P* value (#)
Gestational age (weeks)^a^	33.72 ± 1.64	34.48 ± 1.78	39.23 ± 0.94	0.346	0.395
Birth weight (g)^a^	2027.50 ± 45.23	2096.30 ± 54.89	3233.75 ± 2.29	1.05	0.789
Male sex, n (%) ^b^	4(50)	5(62.5)	4(50)	0.254	0.614
Cesarean section, n (%) ^b^	5(62.5)	4(50)	4(50)	0.254	0.614
Maternal milk predominantly n (%) ^b^	6(75)	5(62.5)	5(62.5)	0.291	0.590
Antibiotic exposure prior to stool sample collection, n (%)	8(100)	8 (100)	-		
Less than 48 h. ^b^	4 (50)	2 (25)	-	1.07	0.302
More than 7 days ^b^	2 (25)	2(25)	-	0	1.0
Total days on antibiotics Before stool sample collection (d)^a^	3.00 ± 2.45	3.63 ± 1.85	-	0.814	0.574
Stool collection time between admission time(d)^a^	3.0 ± 2.45	3.63 ± 1.85	-	0.814	0.574
Age (d)^a^	35.25 ± 18.53	38.75 ± 23.84	-	0.885	0.748
WBC(×10^9^/l)^a^	9.15 ± 5.76	10.59 ± 3.44	-	3.39	0.55
NUET (%) ^a^	45.21 ± 19.53	41.24 ± 19.77	-	0.006	0.69
Plc (×10^9^/l) ^a^	314.75 ± 149.42	478.25 ± 153.58	-	0.067	0.049
CRP (mg/l) ^a^	5.93 ± 6.88	6.03 ± 13.76	-	0.44	0.99
PCT (ng/dl) ^a^	4.41 ± 12.02	0.12 ± 0.08	-	5.35	0.33

Data are expressed as the mean (standard error of the mean) for continuous variables or n (percentage) for categorical variables. Differences between the LOS group and pneumonia group were analyzed using a t test (marked as ^a^) for quantitative variables. The χ^2^ test was used for categorical variables (marked as ^b^). “#” indicates a comparison between the LOS group and the pneumonia group. Abbreviations: d: day, white blood cell (WBC), neutrophil ratio (NR), blood platelet count (Plc), C-reactive protein (CRP), and procalcitonin (PCT)

### Gut microbiota analysis

Twenty-four samples were included in this study. The sample rarefaction curves (Supplementary Fig. 1A) and the Shannon–Wiener curves (Supplementary Fig. 1B) of all the samples supported the adequacy and rationality of the sampling efforts.

#### Gut microbiota characteristics of the LOS group, pneumonia group, and control group

**Fig. 1:**
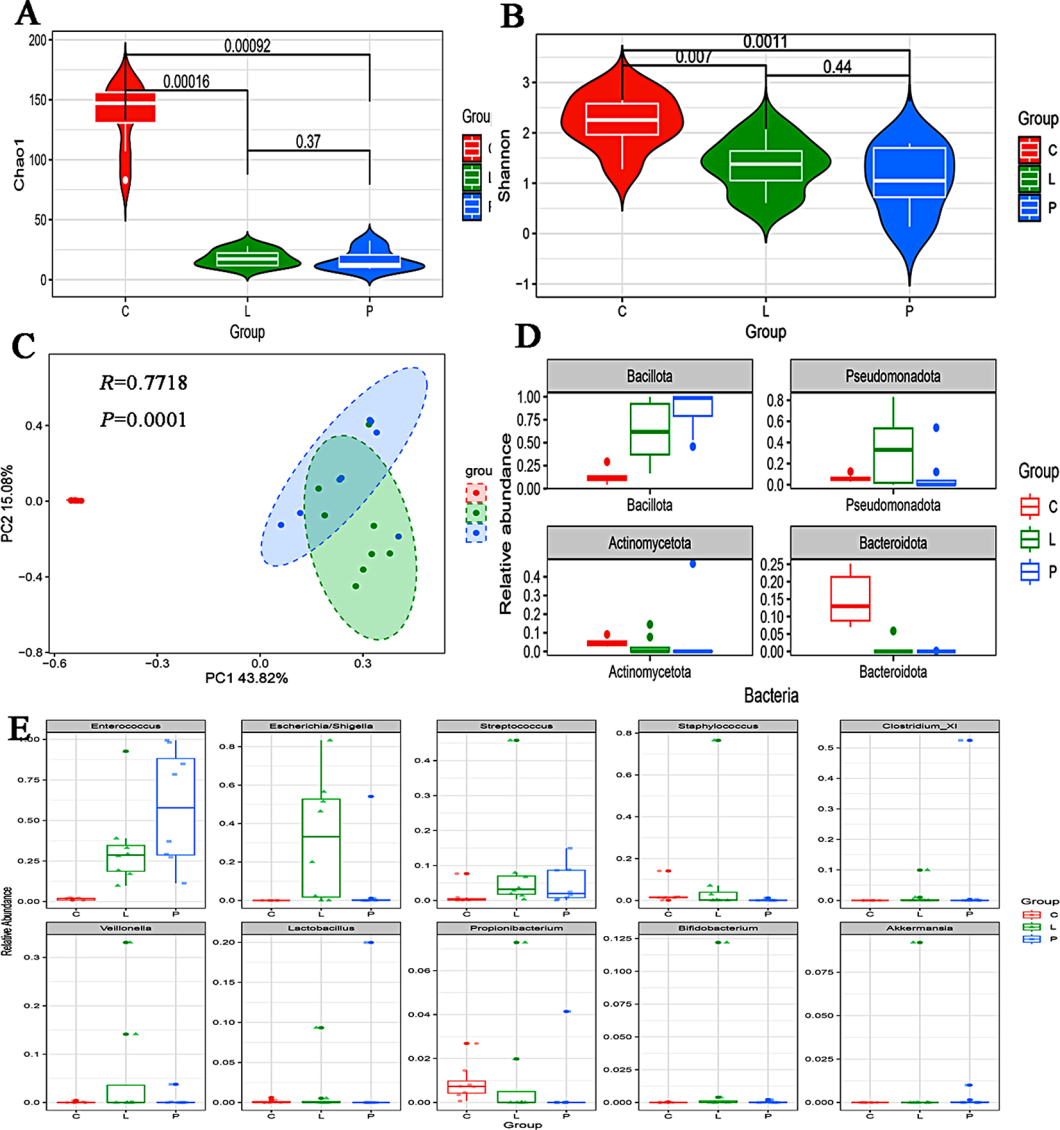
Gut microbiota diversity and relative abundance in the pneumonia, LOS, and control groups. (**A**) Comparison of Chao1 index among the three groups. (**B**) Comparison of Shannon index among the three groups. (**C**) PCoA among the three groups. (**D**) Histogram showing the top 4 phyla by abundance. (**E**) Histogram showing the top 10 genera by abundance. L: LOS group (*n* = 8), P: pneumonia group (*n* = 8), C: control group

There were significant differences in α diversity (Fig. 1A, B, P *<* 0.05) and PCoA (Fig. 1C, ANOSIM, *R =* 0.7718, *P =* 0.0001) results among the three groups.

At the phylum level, there was decreasing trend in the relative abundance of *Pseudomonadota* (0.326 vs. 0.084 vs. 0.061) in the LOS, pneumonia, and control groups (Fig. 1D). At the genus level, the relative abundances of *Escherichia/Shigella* (0.324 vs. 0.068 vs. 0), *Streptococcus* (0.087 vs. 0.046 vs. 0.012) and *Akkermansia* (0.012 vs. 0.001 vs. 0) showed a decreasing trend in the LOS group, pneumonia group, and control groups (Fig. 1E). In the analysis of microbial composition, the LEfSe results revealed distinct dominant microbiota across groups (Supplementary Fig. 2).

#### Gut microbiota characteristics of the control group and LOS group

**Fig. 2:**
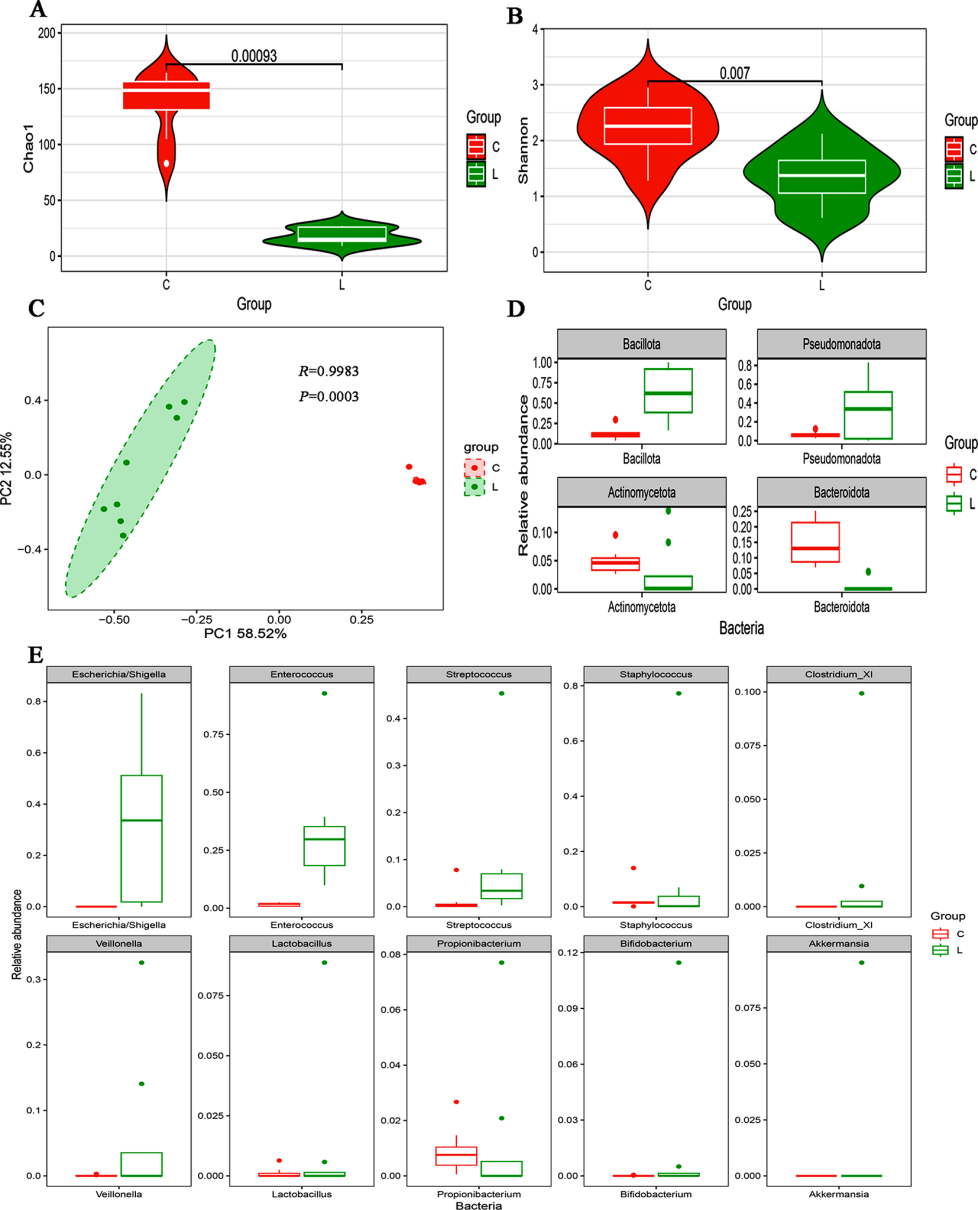
Gut microbiota diversity and relative abundance in the LOS and control groups. (**A**) Comparison of Chao1 index between the two groups. (**B**) Comparison of Shannon index between the two groups. (**C**) PCoA between the two groups. (**D**) Histogram showing the top 4 phyla by abundance. (**E**) Histogram showing the top 10 genera by abundance. L: LOS group (*n* = 8), P: pneumonia group (*n* = 8), C: control group (*n* = 8)

The Chao1 index and Shannon index were greater in the control group than in the LOS group (Fig. 2A, B, P *<* 0.05). The β diversity analysis (PCoA) results revealed significant differences in the intestinal microbiota between the LOS group and the control group (Fig. 2C, ANOSIM, *R =* 0.9983, *P =* 0.0003).

Variations among the taxa were mainly compared at the phylum and genus levels. The predominant phylum in LOS group were *Bacillota*, *Pseudomonadota*. The predominant phylum in control group were unassigned, *Bacteroidota* and *Bacillota*. At the phylum level, the relative abundances of *Bacillota* (0.629 vs. 0.123, *FDR* = 0.006) and *Pseudomonadota* (0.324 vs. 0.062, *FDR* = 0.039) were significantly greater in the LOS group than in the control group, and the relative abundance of *Bacteroidota* (0.0069 vs. 0.15, *FDR* = 0.0019) was significantly lower in the LOS group than in the control group (Fig. 2D). At the genus level, the predominant genera in LOS groups (Fig. 2E) were *Enterococcus*, *Escherichia/Shigella*, *Staphylococcus*, and *Streptococcus*. The major genera in control group were unassigned, *Bacteroides*, *Sphingobacterium*, and *Staphylococcus*. By comparing the two groups (Fig. 2E), the relative abundances of *Enterococcus* (0.339 vs. 0.016, *FDR =* 0.002), *Akkermansia* (0.012 vs. 0, *FDR =* 0.002), *Escherichia/Shigella* (0.323 vs. 0, *FDR =* 0.006), and *Clostridium_XI* (0.013 vs. 0, *FDR =* 0.002) were noticeably greater in the LOS group, while the abundances of *Sphingobacterium* (0 vs. 0.049, *FDR* = 0.002), *Prevotell*a (0 vs. 0.011, *FDR* = 0.002), *Lysinibacillus* (0 vs. 0.007, *FDR* = 0.002), *Faecalibacterium* (0 vs. 0.005, *FDR* = 0.001), *Bacillu*s (0.00002 vs. 0.004, *FDR* = 0.002), *Bacteroides* (0.0032 vs. 0.051, *FDR* = 0.002), and *Stenotrophomonas* (0.001 vs. 0.0059, *FDR* = 0.02) were significantly lower in the LOS group.

Consistent with the pathogenic bacteria in the LOS group, *Bacillota* and *Pseudomonadota* accounted for 95.3% of the pathogenic bacteria in the LOS group at the phylum level. The top 4 pathogenic bacteria at the genus level were determined (Table 2).

#### Gut microbiota characteristics of the control group and pneumonia group

**Fig. 3:**
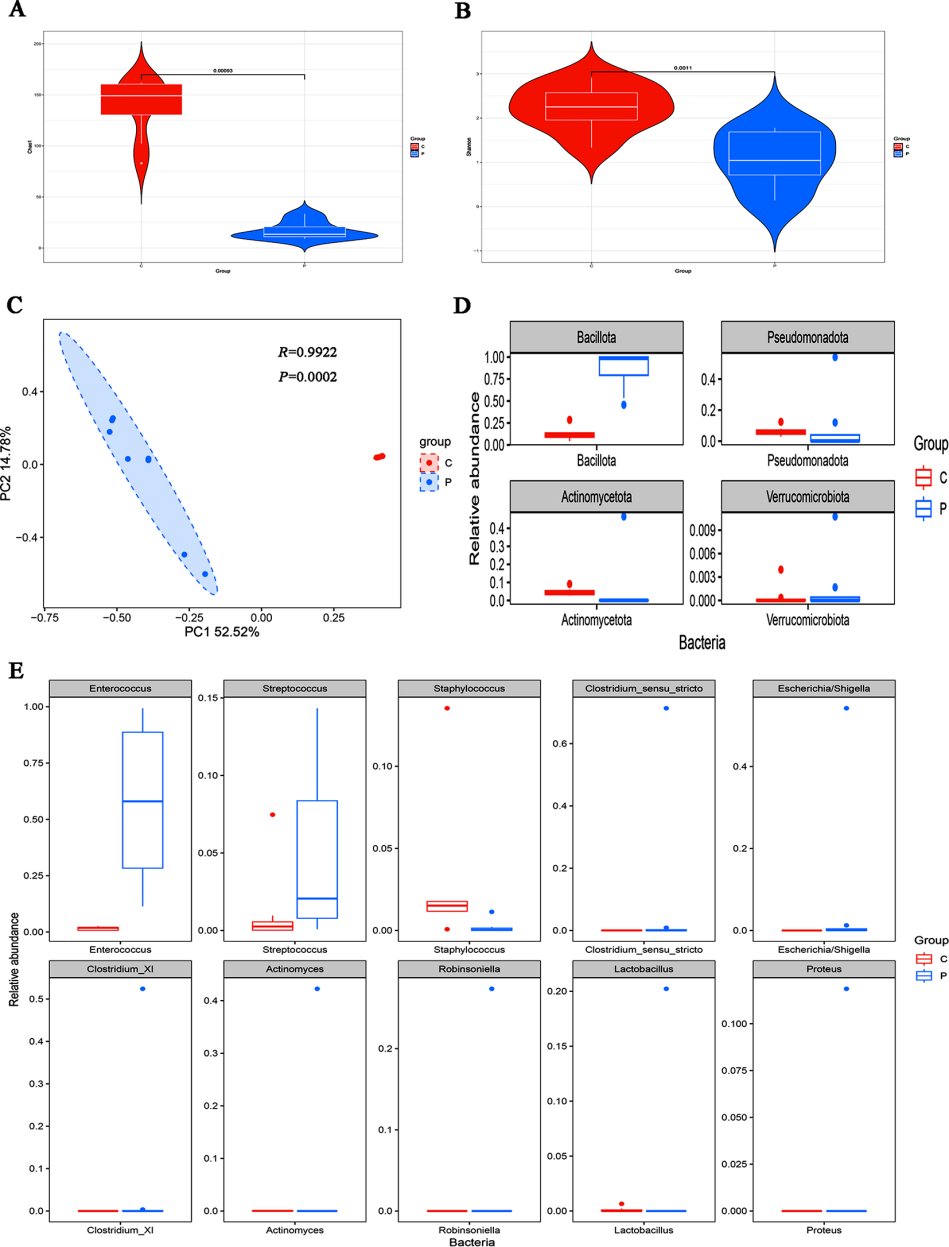
Gut microbiota diversity and relative abundance in pneumonia and control groups. (**A**) Comparison of Chao1 index between the two groups. (**B**) Comparison of Shannon index between the two groups. (**C**) PCoA between the two groups. (**D**) Histogram showing the top 4 phyla by abundance. (**E**) Histogram showing the top 10 genera by abundance. L: LOS group (n = 8), P: pneumonia group (n = 8), C: control group (n = 8)

Metastats analysis showed that the relative abundance of *Bacillota* at the phylum level was considerably greater in the pneumonia group than in the control group (Fig. 3D, 0.8549 vs. 0.1224, *FDR =* 0.0009). In terms of genera, *Enterococcus*, *Robinsoniella*, *Proteus*, *Akkermansi*a, and *Escherichia/Shigella* were significantly more abundant (Fig. 3E, 0.5837 vs. 0.1259, *FDR =* 0.002; 0.0342 vs. 0, *FDR =* 0.002; 0.0148 vs. 0, *FDR =* 0.002; 0.0016 vs. 0, *FDR =* 0.002; 0.0696 vs. 0, *FDR =* 0.005) in the pneumonia group, while *Staphylococcus*, *Ochrobactrum*, *Bacteroides*, *Acinetobacter*, *Flavobacterium*, *Parabacteroides*, and *Alistipes* were significantly less abundant (Fig. 3E, 0.0019 vs. 0.0281, *FDR =* 0.002; 0 vs. 0.0259, *FDR =* 0.002; 0.0002 vs. 0.0518, *FDR =* 0.0038; 0.00003 vs. 0.0018, *FDR =* 0.0038; 0 vs. 0.0107, *FDR =* 0.002; 0 vs. 0.0089, *FDR =* 0.002; 0 vs. 0.0033, *FDR =* 0.002) in the pneumonia group.

#### Gut microbiota characteristics of the LOS group and pneumonia group

**Fig. 4:**
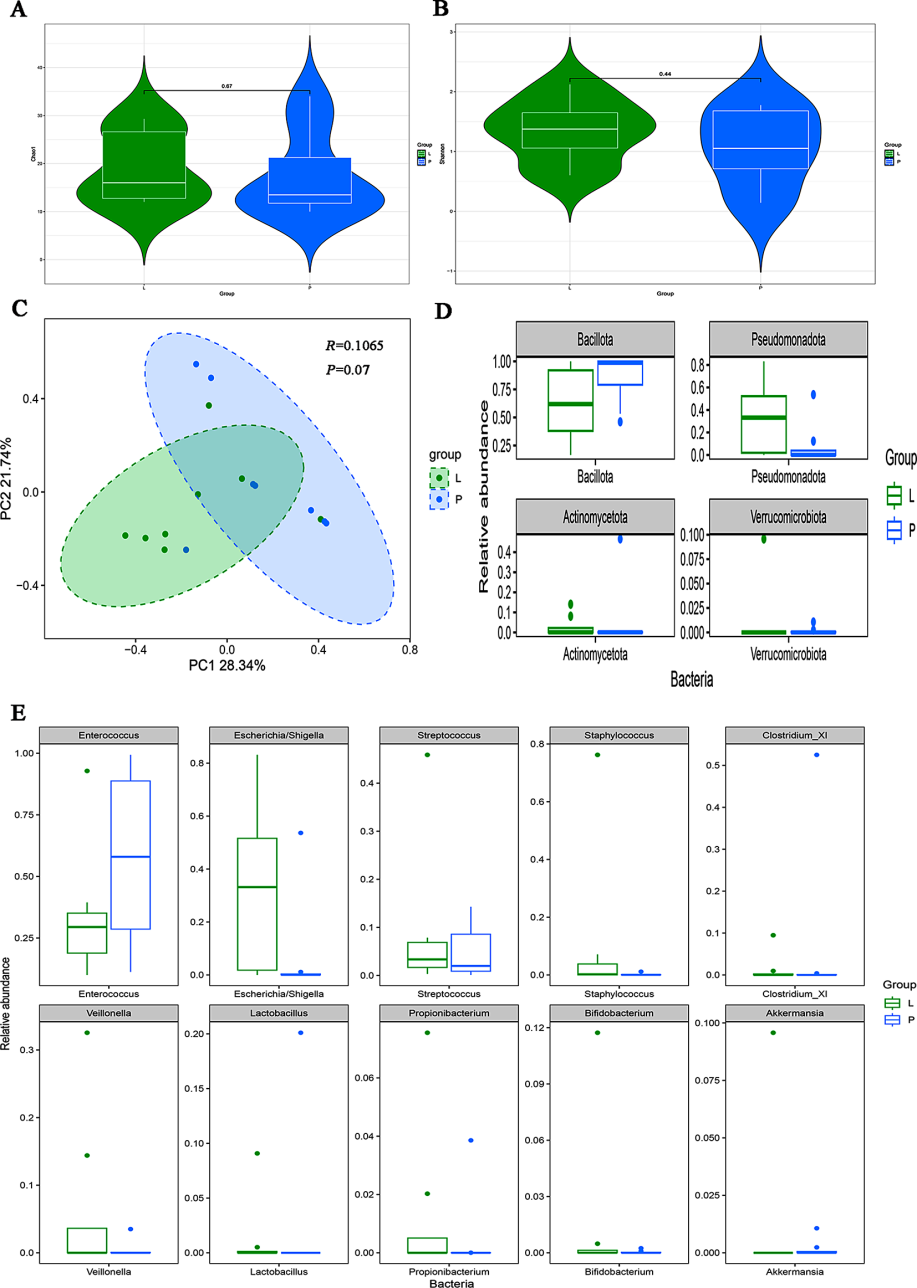
Intestinal microbiota diversity and relative abundance in the pneumonia and LOS groups. (**A**) Comparison of Chao1 index between the two groups. (**B**) Comparison of Shannon index between the two groups. (**C**) PCoA between the two groups. (**D**) Histogram showing the top 4 phyla by abundance. (**E**) Histogram showing the top 10 genera by abundance. L: LOS group (*n* = 8), P: pneumonia group (*n* = 8), C: control group (*n* = 8)

There was no difference in diversity between the LOS group and pneumonia group (Fig. 4A, B, C).

Regarding taxonomic variations, the relative abundance of *Pseudomonadota* in the LOS group was four times greater than that in the pneumonia group (Fig. 4D, 0.32 vs. 0.08, *FDR =* 0.079). At the genus level, when comparing the LOS and pneumonia groups, the abundances of *Aeromonas*,* Actinomyce*s, *Parabacteroides*, *Stenotrophomonas*, and *Escherichia/Shigella* exhibited significant differences, as shown in Fig. 4E and Supplementary Table 2. Except for *Enterococcus*, *Staphylococcus*, *Streptococcus*, and *Akkermansia*, the abundances in the LOS group were greater than those in the pneumonia group, although the differences were nonsignificant, as shown in the Supplementary Table 2.

### Distribution of cultured pathogens

Table 2 presents a compilation of the bacterial isolates responsible for LOS in the included preterm infants. One (12.5%) case had gram-negative bacteria, while the remaining 7 cases (87.5%) had gram-positive bacteria. The neonates with *S. pneumonia* and *Group B Streptococcus* bacteremia were not simultaneously diagnosed with pneumonia. The sputum culture results for the pneumonia group are presented in Supplementary Table 3.

**Table 2:** Primary pathogens in LOS patients and the pathogen profiles

Patient	Blood-Cultured Pathogen	Phylum (%)	Class (%)	Order (%)	Family (%)	Genus (%)
L1	*Escherichia coli*	*Pseudomonadota* (0.05)	*γ-Pseudomonadota* (0.025)	*Enterobacteriales* (0.025)	*Enterobacteriaceae* (0.025)	*Escherichia* (0.025)
L2	*Enterococcus faecium*	*Bacillota* (44.05)	*Bacillibacteria* (34.12)	*Lactobacillales* (34.12)	*Enterococcaceae* (33.82)	*Enterococcus* (33.82)
L3	*Staphylococcus epidermidis*	*Bacillota* (79.8)	*Bacillibacteria*(73.67)	*Bacillales* (73.61)	*Staphylococcaceae*(0.059)	*Staphylococcus*(0.058)
L4	*Streptococcus pneumoniae*	*Bacillota* (89.15)	*Bacillibacteria*(54.44)	*Lactobacillales*(56.15)	*Streptococcaceae*(7.91)	*Streptococcu*s(7.91)
L5	*Staphylococcus warneri*	*Bacillota* (99.88)	*Bacillibacteria*(98.90)	*Bacillales* (77.25)	*Staphylococcaceae*(77.25)	*Staphylococcus*(77.25)
L6	*Enterococcus faecalis*	*Bacillota* (33.53)	*Bacillibacteria*(19.32)	*Lactobacillales*(19.32)	*Enterococcaceae*(17.50)	*Enterococcus*(17.5)
L7	*Group B Streptococcus*	*Bacillota* (16.61)	*Bacillibacteria*(16.60)	*Lactobacillales*(16.59)	*Streptococcaceae*(6.68)	*Streptococcus*(6.68)
L8	*Staphylococcus epidermidis*	*Bacillota* (40.06)	*Bacillibacteria*(40.04)	*Bacillales* (6.96)	*Staphylococcaceae*(6.96)	*Staphylococcus*(6.96)

### Predicted value of LOS risk

**Fig. 5:**
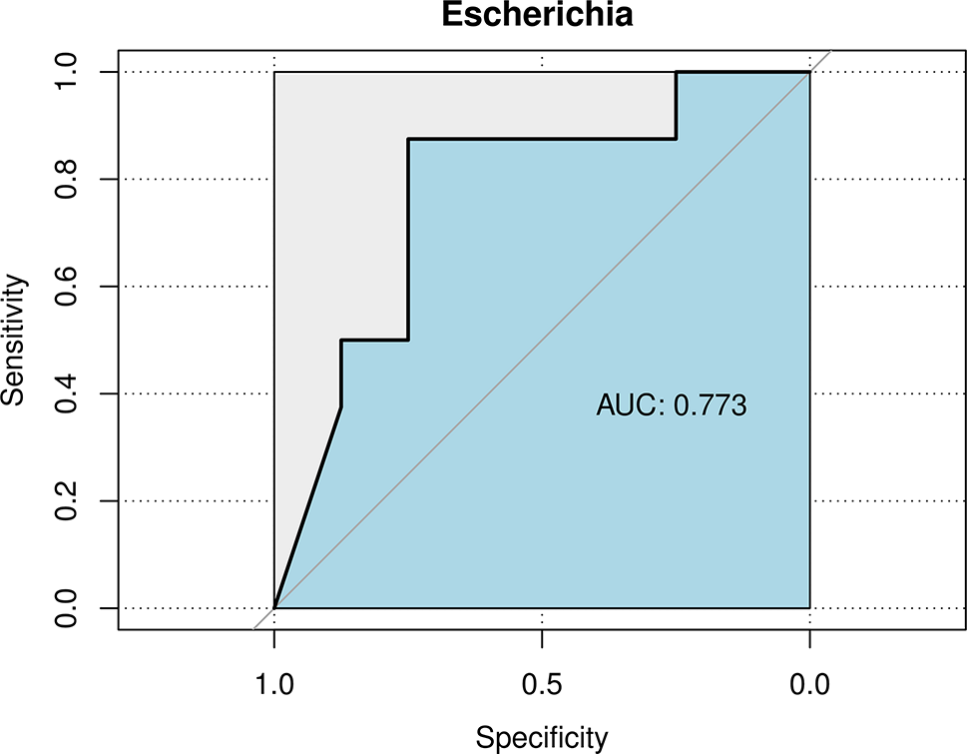
ROC curve of *Escherichia/Shigella* for distinguishing LOS. AUC: Area Under the Curve

The ROC curve was used to evaluate effective LOS biomarkers (Fig. 5). The outcomes demonstrated that there was a difference between the LOS and pneumonia groups. In particular, the AUC of *Escherichia/Shigella* was 0.773 [95% CI (0.521–1), *P =* 0.066], suggesting that an increased abundance of *Escherichia/Shigella* may indicate a greater risk of LOS, potentially serving as a potential biomarker under specific clinical conditions.

## Discussion

In our study, we found that the α diversity and β diversity determined by PCoA were greater in the control group than in the LOS and pneumonia groups. In terms of phylum and genus, we found that an increased abundance of *Escherichia/Shigella*, *Akkermansia*, and *Enterococcus*, as well as a decreased abundance of *Bacteroides* and *Stenotrophomonas*, indicated a high risk for LOS and pneumonia. The pathogenic bacteria causing LOS in infants were consistent with the predominant bacteria found in the gut microbiota. An increased abundance of *Escherichia/Shigella* was associated with a high risk of LOS, with an ACU of 0.7734. The gut microbiota is important in the pathogenesis of LOS and pneumonia. Microbial changes have the potential to serve as early noninvasive biomarkers.

Studies have shown that the intestinal microbiome is crucial for the barrier function of gastrointestinal epithelial cells, and changes in the microbiome can affect intestinal permeability [27]. Furthermore, studies have shown that the development of LOS is related to a decrease in microbial diversity [11]. Our study revealed significant differences in diversity among the LOS group, pneumonia group, and control group. Specifically, the Chao1 index and Shannon index were greater in the control group than in the LOS and pneumonia groups, which is consistent with previous studies. A study reported that mice with greater intestinal microbiota diversity had higher survival rates in the presence of sepsis. Additionally, the survival rate of mice with low diversity also improved significantly when mice with different levels of diversity were reared together. Moreover, the transplantation of intestinal microbiota from sepsis-surviving mice led to a notable increase in the survival rate of susceptible mice [28]. Lankelma et al. also reported that patients with severe sepsis who were admitted to the intensive care unit (ICU) had a low diversity of microbiota structures [29]. The intestinal microbiota of patients with severe pneumonia and recurrent respiratory tract infections is often characterized by low diversity [30, 31]. As a result, the reduced diversity of the intestinal microbiota in premature infants with LOS and pneumonia may increase the risk of illness due to the increasing instability of the microflora [32].

In our study, we found that the relative abundances of *Bacillota* and *Pseudomonadota* were considerably greater at the phylum level in the LOS group and pneumonia group than in the control group, while the relative abundance of *Bacteroidota* was significantly lower in the LOS group and pneumonia group. Interestingly, the relative abundance of *Pseudomonadota* in the LOS group was four times greater than that in the pneumonia group. This study is consistent with relevant literature reports showing that *Bacillota* and *Pseudomonadota* dominate the intestinal microbiota of premature infants with LOS at the phylum level [33]. A previous study compared the differences in intestinal microbiota composition between sepsis patients and healthy people and revealed that the abundance of *Pseudomonadota* in sepsis patients was significantly greater [2]. An increase in the abundances of *Bacillota* and *Pseudomonadota* was also found in a study related to respiratory tract infections [31]. In our study, both the LOS and pneumonia groups demonstrated a decreased abundance of *Bacteroidota* compared to the control group. Other studies have shown similar patterns of bacterial colonization [34]. Therefore, this specific pattern of microbial community changes may be directly associated with an increased risk of these diseases. Our findings provide more concrete evidence for understanding the link between dysbiosis of the microbiome and severe infectious diseases in neonates.

We demonstrated that at the genus level, the most common genera in LOS group were *Enterococcus*, *Escherichia/Shigella*, *Staphylococcus*, and *Streptococcus*, which were significantly correlated with the pathogens causing LOS [35, 36]. In a cohort study with 31/71 infants with sepsis was confirmed by blood culture prior to the diagnosis of LOS, the results showed that there was 93.5% concordance between the pathogen identified in blood culture and that identified in fecal samples [11]. With respect to *Staphylococcus*, recent reports have shown that *Staphylococcal* sepsis is correlated with an overabundance of *Staphylococcus* OTUs in the fecal microbiota [37]. After the occurrence of LOS, gastrointestinal dysfunction, major alterations to the total and proportion of gastrointestinal microbiota, the loss of the obligate anaerobic bacteria (*Bacteroidota*) that typically dominate the intestinal tract in healthy individuals, and mass reproduction of the low-abundance groups (such as *Proteus* bacteria) mass reproduction create conditions for pathogenic bacteria to dominate the intestinal tract [38]. This may indicate that the pathogen causing LOS originates from the intestinal tract.

Compared with those in the control group, the relative abundances of *Enterococcus*, *Escherichia/Shigella*, *Akkermansia*, and *Clostridium_XI* were considerably greater in the LOS and pneumonia groups. In addition, the abundances of *Bacillus*, *Bacteroides*, and *Stenotrophomonas* were significantly lower in the LOS group and pneumonia group. These findings in our study were similar to those of previous studies [33, 37]. Although the pathophysiology of sepsis is multifaceted and poorly understood, disturbance of the intestinal microbiome contributes to sepsis and adversely affects the prognosis of sepsis patients [39]. Pathogen selection (possibly harmful bacteria that may be present in the intestinal microbiome), immunological dysfunction, and decreased production of short-chain fatty acids (SCFAs) (beneficial substances produced by the intestinal microbiome) are frequently triggered by changes in the intestinal microbiome. *Akkermansia* was once considered a new generation of probiotics. However, excessive *Akkermansia* has been associated with the destruction of host mucin, increased intestinal permeability, and the occurrence of inflammation [40]. *Akkermansia* exacerbates intestinal inflammation in *Salmonella typhimurium* [41]. Colorectal cancer is worsened by increased operational taxonomic units (OTUs) of *Akkermansia* [42]. Seibert et al. reported that the abundance of *Akkermansia* significantly increased the risk of allergic disease in mice challenged with a high dose of severe acute respiratory syndrome coronavirus 2 (SARS-CoV-2) virus [43]. The abundance of *Akkermansia* is increased in individuals with *Citrobacter rodentium* infection [44], *Salmonella typhi* infection [45], *Candida tropicalis* infection [46], *rotavirus* infection [47], and graft vs. host disease(GvHD) [48, 49]. *A. muciniphila* has been shown to promote infection by the pathogens *Citrobacter rodentium*, and *Clostridioides difficile* [50, 51]. It is suggested that an increase in the relative abundance of *Akkermansia* may promote the development of infection. We found that the reduction in *Stenotrophomonas* abundance may be related to infection, which may reflect the disruption of the intestinal microbiota. Currently, there is no evidence that *Stenotrophomonas*, which accounts for a large proportion of the microbiota in breast milk [52], is beneficial for infant health. In fact, *Stenotrophomonas* is a potentially pathogenic bacterium that can cause infections in immunocompromised individuals [53]. It is worth noting that *Stenotrophomonas* is not typically considered a beneficial intestinal microorganism, and its presence should be monitored and further studied.

In this study, the abundance of *Actinomyces* was considerably greater in the pneumonia cohort than in the LOS cohort. Similar to the results of Mai et al. [33], this study revealed that the abundance of *Bifidobacteria*, a genus of the phylum *Actinomycetota*, was lower in children who subsequently developed LOS. Taft et al. discovered lower levels of *Actinomycetota* in initial samples from infants who eventually developed LOS than in those from controls [54]. Consistent with earlier investigations, the relative abundances of *Clostridium* and *Lactobacillus* in the pneumonia group were greater than those in the LOS group. *Clostridium*, *Klebsiella*, and *Veillonella* are dominant bacteria in healthy infants [32]. *Clostridium* can produce SCFAs, which are unique nutritional and energy components of the intestinal epithelium, and can improve the regulation of lung immune and inflammatory responses, relieve lung pathology and reduce the occurrence of LOS [55]. Immunological dysfunctions and alterations in the secretion of SCFAs play significant roles in the development and progression of LOS. We plan to use methods such as immunohistochemistry, enzyme-linked immunosorbent assay (ELISA), and metabolomics in our future research to further investigate these aspects.

Our results showed that distinct difference between the LOS and pneumonia groups in the abundance of *Escherichia/Shigella*, which had greatest considerable AUC value of 0.773. The p value was not significant, which may be related to our small sample size. When the relative abundance of *Escherichia/Shigella* in the intestinal tract increases significantly and immunity decreases, the bacteria can reproduce and cause infection. *Escherichia/Shigella* are a group of bacteria whose cell walls contain lipopolysaccharides (LPSs), and the presence of these bacteria can lead to an increase in the total LPS level [56, 57] and induce macrophage death [58]. In reaction to dangerous gut bacteria, the body may carefully control the immune system while tolerating symbiotic microorganisms. The intestinal microbiota cannot be simply divided into pathogenic or nonpathogenic bacteria. Many native gut bacteria can cause disease when conditions are conducive. For example, *Escherichia coli* can induce sepsis, *Bacteroides* can induce abscesses, *Enterococcus* can induce endocarditis, and *Clostridium histolyticum* can induce gas gangrene. The gut microbiota is normally kept in check by a thick mucus layer, an intact epithelial barrier, and immune cells. However, under certain circumstances, these barriers can be compromised. Patients with sepsis often experience changes in their intestinal physiology as a result of inherent factors (systemic inflammation and epithelial permeability) or extrinsic factors (antibiotic use and parenteral feeding). These conditions can potentially alter the gut microbiota and the intestinal barrier, consequently increasing the risk of bacterial infection and sepsis [59, 60]. It is important to note that this dysbiosis may reflect, rather than directly cause, infant susceptibility to LOS.

This study has several limitations. The first limitation is the small number of samples collected in the LOS and pneumonia groups. Although statistical significance was achieved for several parameters in this study, in the future, a larger number of samples would be beneficial to confirm our findings. Another limitation was that feces were collected during diagnosis. Collecting the infants’ feces during diagnosis did not allow us to identify which microbes may have predisposed the infants to infection. Additionally, the infants had already received several different antibiotics before stool sample collection (the average treatment time was less than four days), which may have influenced the results. Fourth, we did not record the duration of fasting, the use of parenteral nutrition, or any other comorbidities of the preterm newborns, which could affect the interpretation of our findings. Finally, the inclusion of healthy full-term infants rather than preterm infants as controls may influence the results.

## Conclusion

Preterm infants with sepsis and pneumonia exhibit decreased diversity and gut microbiota disturbances. Our results showed that the dominant bacteria in the intestinal microbiota of the LOS group were significantly related to the pathogen causing LOS. The increased abundance of *Escherichia/Shigella* in preterm infants may reflect the risk of LOS occurrence. The intestinal microbiota of premature infants may differ in different hospitals or regions, so it is essential to increase the sample size and conduct more large-sample, multicenter, randomized controlled studies in the future.

### Electronic supplementary material

Below is the link to the electronic supplementary material.

Supplementary Material 1

Supplementary Material 2

## Data Availability

The data presented in the study are deposited in the National Library of Medicine (NCBI) repository (accession number PRJNA1040967).

## Abbreviations

LOS: Late-Onset Sepsis
LEfSe: Linear Discriminant Analysis Effect Size
AUC: Area Under The Curve
I/T: Immature To Total
WBC: White Blood Cell
NR: Neutrophil Ratio
Plc: Blood Platelet Count
CRP: C-Reactive Protein
PCT: Procalcitonin
ACE: Abundance-Based Coverage Estimator
PCA: Principal Component Analysis
PCoA: Principal Coordinates Analysis
LDA: Linear Discriminant Analysis
NMDS: Nonmetric Multidimensional Scaling
ICU: Intensive Care Unit
OTUs: Operational Taxonomic Units
SCFAs: Short-Chain Fatty Acids
LPS: Lipopolysaccharide
