# Supplementary material for: Gut microbiota in preterm infants with late-onset sepsis and pneumonia: a pilot case-control study
Source: BMC Microbiol. 2024 Jul 22;24:272. doi: 10.1186/s12866-024-03419-w (PMC11265154; doi:10.1186/s12866-024-03419-w)
Supplement: Supplementary file 1 — Supplementary Material 1 [file 12866_2024_3419_MOESM1_ESM.docx]

**Supplemental Tables and Figures**

**Supplemental Tables**

Supplementary Table 1 Details of Antibiotic Usage

| Variables | Preterm infants with LOS,  N = 8 | Preterm infants with pneumonia,  N = 8 |
| --- | --- | --- |
| Antibiotic exposure, yes, n (%) | 8(100) | 8(100） |
| Antibiotic exposure prior to fecal sample collection n (%) | 8(100) | 8(100) |
| Less than 24 hrs, n (%) | 3 (37.5) | 1 (12.5) |
| Less than 48 hrs, n (%) | 1 (12.5) | 1 (12.5) |
| About 3-5 days, n (%) | 2 (25) | 4 (50) |
| More than 5 days, n (%) | 2 (25) | 2（25） |
| Total days on antibiotics before fecal Sample collection (days) | 3.00 ±2.45 | 3.63±1.85 |
| Types of antibiotics before stool specimen |  |  |
| Penicillin, n (%) | 1 (12.5) | 3 (37.5) |
| Cefotaxime, n (%) | 4 (50) | 0 |
| Cefepime, n (%) | 0 | 1(12.5) |
| Moxalactam, n (%) |  | 1 (12.5) |
| Piperacillin-tazobactam, n (%) | 2（2.5） | 2 (25) |
| Meropenem, n (%) | 1 (12.5) |  |
| Metronidazole, n (%) | 0 | 1(12.5) |

Data are expressed as n (percentage) for categorical variables. In both the LOS group and the pneumonia group, 8 patients each had used antibiotics prior to the collection of fecal samples. In the LOS group, 3 patients had used antibiotics for less than 24 hours, 1 patient for less than 48 hours, 2 patients for 3-5 days, and 2 patients for more than 5 days, with an average duration of antibiotic use being 3.00 ± 2.45 days. The types of antibiotics used included penicillin in 1 case, cefotaxime sodium in 4 cases, piperacillin-tazobactam in 2 cases, and meropenem in 1 case. In the pneumonia group, 1 patient had used antibiotics for less than 24 hours, 1 patient for less than 48 hours, 4 patients for 3-5 days, and 2 patients for more than 5 days. The types of antibiotics used included penicillin in 3 cases, cefepime in 1 case, moxalactam in 1 case, piperacillin-tazobactam in 2 cases, and metronidazole in 1 case.

Supplementary Table 2 Primary Pathogens in pneumonia patients and pathogen profiles

| Patient | Sputum-Cultured Pathogens | Phylum (%) | Class (%) | Order (%) | Family (%) | Genus (%) |
| --- | --- | --- | --- | --- | --- | --- |
| P1 | *Klebsiella pneumoniae* | *Pseudomonadota*（0.17%） | 0 | 0 | 0 | 0 |
| P2 | *Staphylococcus aureus* | *Bacillota*（87.70%） | *Bacilli*（87.3%） | *Bacillales*（0.18%） | *Staphylococcaceae*（0.18%） | *Staphylococcus*（0.18%） |
| P3 | *Klebsiella pneumoniae* | *Pseudomonadota*（1.29%） | *Gamma-proteobacteria*（1.12%） | *Enterobacteriales*（1.11%） | *Enterobacteriaceae*（1.1%） | 0 |

Three pathogens identified as clearly pathogenic in the pneumonia (P) group, 2 cases of *Klebsiella pneumoniae*, 1 case of *Staphylococcus aureus*.

Supplementary Table 3 Different microbiota between the LOS and pneumonia groups.

| Taxon | P_mean | P_variance | L_mean | L_variance | *p*_value |
| --- | --- | --- | --- | --- | --- |
| *Aeromonas* | 0 | 0 | 0.0001 | 0 | 0.001 |
| *Actinomyces* | 0.0530 | 0.0225 | 0 | 0 | 0.001 |
| *Parabacteroides* | 0 | 0 | 0.004 | 0.0001 | 0.001 |
| *Stenotrophomonas* | 0 | 0 | 0.001 | 0.000009 | 0.001 |
| *Escherichia/Shigella* | 0.0694 | 0.0362 | 0.325 | 0.0973 | 0.045 |
| *Staphylococcus* | 0.0019 | 0 | 0.108 | 0.07 | 0.09 |
| *Enterococcus* | 0.584 | 0.127 | 0.339 | 0.066 | 0.165 |
| *Streptococcus* | 0.046 | 0.0027 | 0.088 | 0.023 | 0.672 |
| *Lactobacillus* | 0.025 | 0.005 | 0.012 | 0.001 | 0.752 |
| *Akkermansia* | 0.00002 | 0 | 0.00012 | 0.00001 | 0.836 |

L: LOS group (n = 8), P: pneumonia group (n = 8).

**Supplemental Figures**


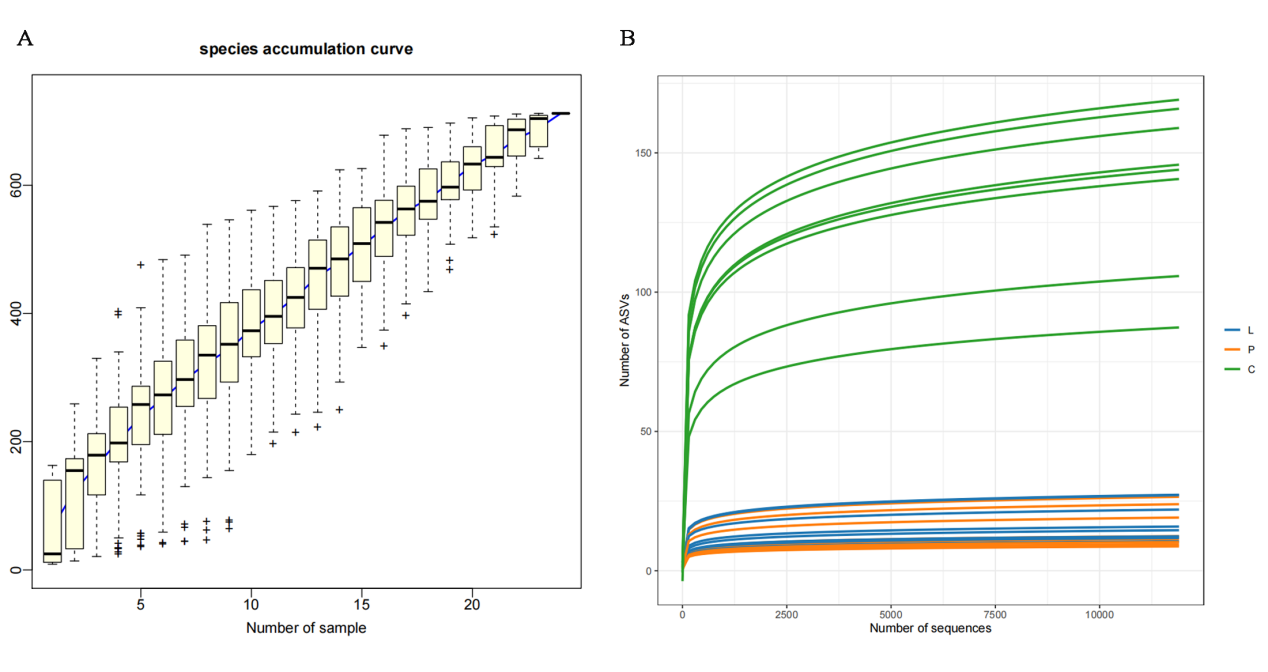
Supplementary Figure 1

Sample curve analysis. (A) Sample rarefaction curves. (B) Sample Shannon–Wiener curves.


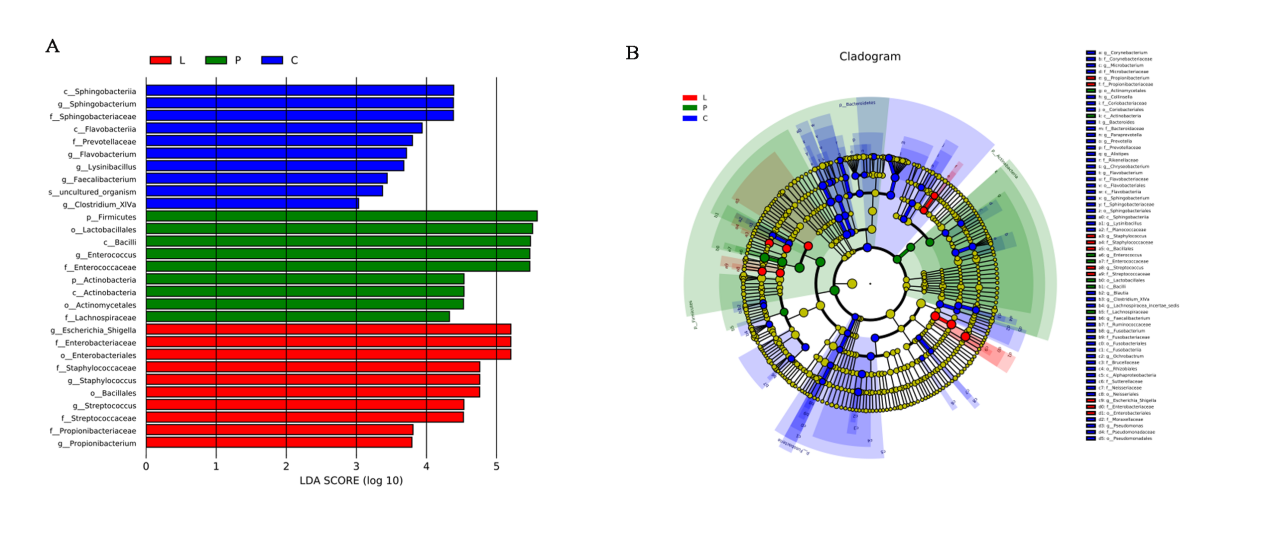


Supplementary Figure 2

LEfSe analysis in the pneumonia, LOS, and control group. (A)Histogram of LDAscores. Demonstrates distinct variations in microorganism type and abundance. LDA scores on a log 10 scale are shown at the bottom. Red for the LOS group, green for the Pneumonia group, and blue for the control group. The length of the bars indicates the contribution size of the differential species (i.e., the LDA Score). The chart displays species that show significant differences in abundance between groups when the LDA Score exceeds a set threshold (default set at 2), identifying biomarkers whose abundance is significantly higher within each group compared to other groups. (B) Cladogram Generated by LEfSe. This is a phylogenetic branching diagram of differential species, with concentric circles radiating from the center to the outer edges representing taxonomic levels from phylum to genus. Each small circle at different taxonomic levels represents a classification at that level, with the circle's diameter proportional to its relative abundance. The coloring principle is as follows: Species with no significant differences are uniformly colored yellow. Differential species are colored according to their group, with red nodes indicating microorganisms playing a significant role in the LOS group, green nodes for those significant in the Pneumonia group, and blue nodes for those significant in the control group. The full names of species represented by English letters in the diagram are displayed in Legend Supplementary Figure 2B. L: LOS group (n = 8), P: pneumonia group (n = 8), C: control group (n = 8). LEfSe: LDA effect size. LDA: Linear discriminant analysis.
