# Supplementary material for: Gut microbiota in preterm infants with late-onset sepsis and pneumonia: a pilot case-control study
Source: BMC Microbiol. 2024 Jul 22;24:272. doi: 10.1186/s12866-024-03419-w (PMC11265154; doi:10.1186/s12866-024-03419-w)
Supplement: Supplementary file 2 — Supplementary Material 2 [file 12866_2024_3419_MOESM2_ESM.docx]

**Supplementary Methods**

Instructions for Fecal Collection

| Refer to the usage diagram | Operational steps |
| --- | --- |
| 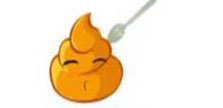  Collect feces from the middle section, inner side. | Wear gloves and use a sterile collection spoon to sample the feces from the diaper. Typically, sample the middle to rear portion of the excreted feces, and sample from the inner middle part of the feces, avoiding sampling from the surface layer to prevent changes in the microbial species on the surface of the feces. |
| 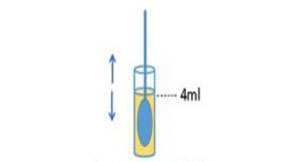  Shake the spoon up and down.  . | During sample collection, generally take a fecal sample approximately the size of a peanut. Place the collection spoon containing the fecal sample into a preservation tube filled with preservation solution. Shake the spoon up and down to ensure the sample is fully immersed in the preservation solution. Ensure that the liquid level of the preservation solution does not exceed 4ml. Remove the collection spoon and tighten the preservation tube lid. Apply the barcode label to the preservation tube. (Note: Approximately 1g of feces requires about 3ml of fecal microbiota preservation solution at room temperature.) |
| 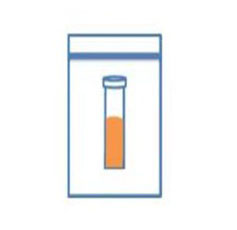 | After collection is completed, we place the preservation tube containing the fecal sample and fecal microbiota preservation solution in a -80℃ freezer for freezing preservation within 30 min. |

After collecting the stool, the steps for homogenization include:

1. **Sample Mixing:** Place the entire stool sample into an appropriate container and mix it thoroughly using a stirrer to ensure thorough blending of all parts of the sample.
2. **Sample Partitioning:** If the sample volume is too large, it can be partitioned into smaller portions, which are then homogenized separately to ensure each portion is adequately processed and analyzed.
3. **Mechanical Homogenization:** Utilize a mechanical homogenizer to homogenize the mixed sample further, ensuring even distribution of microbes and other constituents throughout the sample.
4. **Time and Speed Control:** Control the homogenization time and speed to ensure that the sample is adequately processed without causing excessive disruption or loss.
5. **Temperature Control:** Pay attention to temperature control during homogenization to prevent adverse effects or damage to the sample.

By following these steps, the stool sample can be homogenized effectively before use, thereby improving the accuracy and reliability of subsequent analyses.
